# Information sharing with rural family caregivers during care transitions of hip fracture patients

**DOI:** 10.5334/ijic.1195

**Published:** 2014-06-16

**Authors:** Jacobi Elliott, Dorothy Forbes, Bert M. Chesworth, Christine Ceci, Paul Stolee

**Affiliations:** School of Public Health and Health Systems, University of Waterloo, Waterloo, Canada; Faculty of Nursing, University of Alberta, Edmonton, Canada; Faculty of Health Sciences, Western University, London, Canada; Faculty of Nursing, University of Alberta, Edmonton, Canada; School of Public Health and Health Systems, University of Waterloo, Waterloo, Canada

**Keywords:** hip fracture, rural health care, family caregivers, ethnography, continuity of care

## Abstract

**Introduction:**

Following hip fracture surgery, patients often experience multiple transitions through different care settings, with resultant challenges to the quality and continuity of patient care. Family caregivers can play a key role in these transitions, but are often poorly engaged in the process. We aimed to: (1) examine the characteristics of the family caregivers’ experience of communication and information sharing and (2) identify facilitators and barriers of effective information sharing among patients, family caregivers and health care providers.

**Methods:**

Using an ethnographic approach, we followed 11 post-surgical hip fracture patients through subsequent care transitions in rural Ontario; in-depth interviews were conducted with patients, family caregivers (*n* = 8) and health care providers (*n* = 24).

**Results:**

Priority areas for improved information sharing relate to trust and respect, involvement, and information needs and expectations; facilitators and barriers included prior health care experience, trusting relationships and the rural setting.

**Conclusion:**

As with knowledge translation, effective strategies to improve information sharing and care continuity for older patients with chronic illness may be those that involve active facilitation of an on-going partnership that respects the knowledge of all those involved.

## Introduction

Hip fractures are a common and serious injury for older persons, with significant adverse consequences for mobility, functional independence and quality of life [1–4]. For hip fracture patients, treatment and recovery are often complicated by other health problems and comorbidities [4]. Following hip fracture surgery, patients often experience multiple transitions through different care settings; these transitions present challenges to the quality and continuity of patient care, and to patient safety [5–7]. Older adults are more susceptible to receiving fragmented care as they move between various health care settings during their rehabilitation journey [8, 9]. Information sharing between care settings is critical to ensure smooth transitions, but is often difficult or ineffective due to inadequate electronic information systems, unclear roles and responsibilities, large ‘circles of care’ within and between care settings and other constraints [5–10].

Often, the only common factor at each transition between health care settings, in addition to the patient, is the family caregiver [11]. Family caregivers have a key role in supporting care transitions, but are often poorly engaged in the process – either as sources or as recipients of important information [12]. Unfortunately, little is known about family caregivers’ experiences during care transitions and the factors that facilitate or hinder the effective sharing of information.

We aimed to enhance our understanding of the role caregivers play during care transitions for hip fracture patients, including: (1) caregivers’ experiences of information sharing during care transitions and (2) facilitators and barriers of effective information sharing and communication.

## Methods

This study was conducted as part of a larger, multi-site Canadian study of care transitions for hip fracture patients (www.inforehab.uwaterloo.ca); other findings have been reported elsewhere [5, 10, 13–15]. In all study sites, hip fracture patients were recruited in acute care post-surgery and followed through resulting transitions throughout their care trajectory until their arrival in their long-term destination (e.g. return home, long-term care placement). Data collection for this paper was conducted in a rural community near London, Ontario.

The study used a qualitative focused ethnographic [16] approach, which is ‘designed to describe and interpret the experiences of research participants in a context-specific setting’ [17, p. 128]. A constructivist–interpretivist stance [18] was taken throughout the research. The main component of this theoretical position is the emphasis on understanding the participants’ experiences. Findings are constructed jointly between the researcher and the participants through the interview questions, from conversational dialogue and interpretation of the data.

Ethics clearance for this project was granted by The University of Western Ontario and University of Waterloo Research Ethics Boards.

### Sample

Purposive sampling (December 2009–January 2011) was conducted to ensure a variety of participants, and post-surgical care settings were included in the study. Patients who were undergoing surgery after a hip fracture, and were over the age of 65, were eligible to be included in the study. Patients with cognitive impairment were also eligible to be included, provided that their next of kin gave informed consent. If there was any indication that there was cognitive impairment, ‘assent’ was requested prior to each interview. Family caregivers were included in the study if they were identified as being involved in the care of the patient. At each care transition point, health care providers who were involved in the patient's discharge or admission (usually two providers at each transition) were recruited. While the focus of this study was on patients and their family caregivers, health care provider data were included because they participated in the communication dynamic during care transitions.

Participants were recruited at a hospital in south-western Ontario. This hospital had two floors (inpatient and outpatient), an emergency room, an operating room and no rehabilitation unit. A research collaborator, a physiotherapist, employed at the hospital acted as a ‘gatekeeper’ and approached potential participants. After the potential participants were approached, they signed a “Consent to be Contacted” form if they were interested in finding out more about the study. The information was passed on to the researchers who then contacted the patients to arrange to meet them in person. A letter of information was then given with an in-depth explanation of the study. In total, 11 patients (mean age 80.4; eight females), eight family caregivers (mean age 57.5) and 24 health care providers consented to participate. Some of the patients (*n* = 6) lived at home with a family member (spouse or adult child); five lived alone prior to their fracture. Of the eight family caregivers enrolled in the study, six were adult children and two were spouses. Table 1 gives a description of the patients, their relationships and the transitions they experienced. Our aim was to interview the patient, a family member and two health care providers at each transition. Following hip-fracture surgery the researchers conducted the first interview with the patient, family caregiver and health care providers involved in the patients’ care. Once the patient was discharged, there was a transition point to another setting (home, long-term care, retirement home, another hospital). The patient, family caregiver and health care providers were interviewed at the new location, asked questions about hospital discharge and about their admission to the next setting (refer to Table 2 for sample interview questions). Because of the small number of health care providers in the rural setting, health care providers may not have been interviewed at all transition points for every patient.

### Data collection

Data were collected through in-depth semi-structured interviews and observations. The interviews were held in private places, such as offices or patient homes, and ranged from 30 to 45 minutes in length. The majority of the interviews were conducted face-to-face, but three (with distant caregivers) were done over the telephone. Interview guides were used as a foundation for the discussion but questions were open-ended in nature and allowed participants an opportunity to expand on a thought or idea wherever necessary. Three interview guides were used, one directed toward the patient, one for the family caregiver and one for the health care provider. The patient interview guide addressed their experiences during admission to hospital, discharge from hospital and returning to home. The health care provider interview guide asked questions regarding what information they generally send or receive when transferring a patient, as well as whether or not they include family caregivers as a source of information. The family caregiver guide explored how they assist and support the patient and the kinds of information they needed and received about the patient's condition during transitions. The interviews were completed at the various locations along the rehabilitation journey. In total, 58 interviews were completed. Interviews were recorded using a digital recorder and transcribed verbatim.

Field notes of observations were completed prior to and following the participant visits to complement interview transcriptions. The use of field notes provided additional information about the non-verbal exchange of information between patients, caregivers and health care providers as participants transitioned between various health care settings. The notes captured behaviours of the patients, family caregivers and health care providers, as well as the interaction among all three parties. Events such as rehabilitation exercises and the discharge process were also observed and recorded on observation forms. During these periods of observation, the researchers gained a sense of the culture among the health care staff, families and patients in the various facilities. They also allowed the researchers to better understand the environment where the information exchanges took place, who was initiating the conversations and non-verbal behaviours. Over the course of the data collection period, 65 hours of observation time was recorded.

Medical documents relevant to participants’ care and transfers within and between each health care setting were also collected, scanned and analysed, providing important additional information about the participants. The three sources of data, face-to-face interviews, observations, and document review, provided the opportunity for triangulation of the data. Data collection ceased when saturation was achieved.

### Data analysis

Transcribed interviews, observation field notes and relevant documents were entered into a qualitative data management programme, NVivo 8 [19], to facilitate thematic analysis. Combining the interviews, observational notes and documents provided depth and allowed the researcher to understand the experiences and processes from all perspectives (caregiver, health care provider and patient). The analysis followed an inductive approach; researchers allowed the themes to emerge directly from the data [20]. Line by line coding was completed by two researchers across all data collected. The codes were grouped into general categories with shared content/aims [21, 22]. The primary data source for analysing caregiver roles and contributions to knowledge exchange were a data subset of eight caregiver interviews and observation notes specific to observing caregiver roles and knowledge exchange. Pertinent references to caregivers made by health care providers and patients, or found in health care documents, were also incorporated into the data analysis.

Several strategies were applied to ensure trustworthiness of the findings [23]. These strategies included peer debriefing [22], consisting of describing the collection of data and analysis processes to the larger InfoRehab group and an audit trail describing the progression of events and interpretations in the study. Triangulation was accomplished through multiple methods of data collection including field notes, interviews and document reviews.

## Results

Families played a major role in providing the health care provider with information such as past medical history, previous ability to complete activities of daily living and other information. In cases where the patient has difficulty communicating, or in the frequent case when information is not received by a health care provider in the new setting from his or her counterpart in the previous setting, families are vital for ensuring this information is obtained.

In most cases, as shown in the excerpt below, family caregivers were viewed by health care providers as a good source of information. This was especially true if the patient was unable to give that information themselves, or if the health care provider did not receive adequate information from the previous care setting.

Interviewer: ‘And does the caregiver ever provide you with information?’

Health care provider: ‘Of course. Again in this case very valuable.’

The following excerpt shows important pre-fracture information that family caregivers can provide about the home-living situation.

Health care provider: ‘Just basically what they've done at home, some people's families will tell us that “yes, they were independent, mobile at home on their own”, other families will say “no they didn't do much walking or they went from a bed to a wheelchair” and that's basically it, they basically tell us about their ADLs [activities of daily living] at home’.

A physiotherapist noted the important contributions that family caregivers make by helping patients with their exercises:

Health care provider: ‘I always try and get the family involved. In this case the wife was very interested.’

Conversely, some health care providers suggested that working with the family caregiver is too difficult – they would much rather just deal with the patient. Sometimes health care providers suggested that family support was lacking and in some cases the family was viewed as ‘useless’ when asked about the amount that families help them to do their job.

Interviewer: ‘OK, and do family members make your work more difficult?’

Health care provider: ‘Sometimes they hinder it and you have to ask them to please leave the room.’

Some health care providers believed that sometimes an inability to be helpful was due to the stressful situation that the family is experiencing:

Interviewer: ‘In general, do family members ever make your work more difficult?’

Health care provider: ‘Um, they can. And it's not always their fault. It's sometimes that they're anxious or maybe they haven't had a chance to talk to the doctor or maybe they just don't understand.’

Guided by our two research aims, multiple sources of data revealed important themes which reflect key characteristics of information sharing and the facilitators and barriers to effective information sharing and communication. Three priority dimensions of information sharing were identified – trust and respect, involvement and information needs – as well as the facilitators and barriers of communication and information sharing, including prior health care experience, trusting relationships and the rural setting; these are described in the following sections.

### Characteristics of positive and negative information-sharing experiences

#### Trusting and respecting health care providers

Themes of trust and respect surfaced frequently in many interviews with caregivers, health care providers and patients. When a family caregiver began to trust the health care providers, they were more comfortable asking questions, making decisions and taking advice from the care providers, as revealed by this caregiver:

Caregiver: ‘She [nurse] was wonderful. I can't thank her enough. She was very good with mother.… you know, we involved mother in everything we said or did, we didn't go behind her back or anything, and she asked us first, you know which way we wanted to go, and we thought mother should be involved, and listen to everything. And she would speak directly to mother and look at us for confirmation, you know, whatever she responded with, you know. I found that really wonderful.’

When family caregivers trusted and respected the health care provider, transitions between health care settings and the transition back home seemed to be less of a burden on family caregivers. However, only a small number of participants described a feeling of trust and respect with the health care providers who were giving care to their family member. On multiple occasions, patients and family caregivers expressed frustration with the staff and were disappointed with their lack of information sharing. For instance, family members found it tiring to chase after health care providers to get answers regarding the care of their family member.

#### Being involved in information sharing

Family caregivers had expectations that they should be involved at all points of the rehabilitation journey. For families, knowing they are involved in the exchange of knowledge and decision making, and being aware of what is happening with their family member is extremely important:

Interviewer: ‘So you felt like you were involved in the decisions, and that you were able to make mum be involved in the decisions?’

Caregiver: ‘Yes. That was definitely a bonus this time, we were very involved.’

However, many caregivers questioned if they had been given adequate information and opportunity to fully participate in the decision-making process regarding the placement of their family member.

Caregiver: ‘You know we would have liked to at least compared you know what could we do for her at home if we have someone come in you know three times a week to bathe her … and this and that. We could help feed her. We were already cooking all her meals anyways towards the end there before she fell.’

Some patients also felt disconnected from the decision-making process. For example, one patient expressed his lack of involvement in decision-making when it came to where he could afford to live following his hospitalization:

Patient: ‘…how to be involved … is them being a little more caring where you're going … you know … like not everybody has the money to go to these places…’

Being involved in the exchange of knowledge and decision-making was particularly difficult for family caregivers who were not able to be at the hospital frequently or at all, and who may have lived at a significant distance. For example, when caregivers were contacted to have a family meeting with health care providers to discuss ‘next steps’, no option was provided for the date and time. They were told when they had to be at the hospital. There was no flexibility, as described by the following caregiver:

Caregiver: ‘They kind of hit us with this one morning we were told to come in and have a meeting with the doctor. It was at 8 o'clock on a Wednesday morning or something’

Interviewer: ‘And there was no flexibility with that meeting?’

Caregiver: ‘None’

Patients also expressed frustration with the inflexibility of health care providers when times were being arranged for a family meeting to discuss the patient's options:

Interviewer: ‘OK, and how about your family? Was your family involved in the discussion?’

Patient: ‘Well, somehow or other they were notified. I wish they wouldn't leave it to me to, because she said Wednesday morning at 8 or 8:30 we're to have a meeting, and my family representative and whoever else, I didn't think I was supposed to be involved. We're to be at this meeting at 8 or 8:30 in the morning! Well I thought holy smoke, my girls just can't drop everything, and 8:30 in the morning, as a matter of fact I was still eating my breakfast.’

Once the decision had been made to transfer the patient, some patients were told what the decision was, without much choice:

Interviewer: ‘Yeah. So were you involved in the decision of where to go next, after the hospital?’

Patient: ‘… they just said it would be either here or [the other hospital]’

Similar experiences occurred when patients were going to be discharged home. Often, they were not asked if they wanted to go home, or if they would be comfortable going home, they were just told that they were being discharged home.

Interviewer: ‘OK, and were you involved in the decision to go home?’

Patient: ‘no, no they just told me.’

Memo: April 16^th^, 2010

‘After the interview was over a doctor came into the room to check on her incision. He told her she was going home today (Friday). She became very anxious - she did not feel ready, her daughter wasn't bringing clothes until the weekend, and she had no support at home. Her husband is blind and can't help her. CCAC told her that she would not be going home until next week.’

The field note illustrates the anxiety that can be caused for patients when important information is being communicated with them with limited understanding of their situation and limited communication and coordination with other health care providers.

#### Family caregiver informational needs and expectations

Throughout the document review, observations at the settings and interviews with caregivers, it was evident that caregivers required additional information to adequately support and care for their family member throughout the transition. Caregivers expressed the need for additional information on topics such as rehabilitation procedures/techniques, what to expect when their family member returns home, what aides should be in place before the patient is discharged from the hospital, what medications will be needed, what home care services are available and whom to call for assistance after discharge.

Caregiver: ‘You know, I mean there were several people [health care providers] who were telling us they have to have an assessment done at home *before* (emphasis) he came home, and then others telling us no, the assessment is done *after* (emphasis)… Now how is someone supposed to come home and be sure they've got what they need?’

Interviewer: ‘And so it didn't happen that way for you?’

Caregiver: ‘No it didn't happen and it really *should* (emphasis) because they need to know what you're going [home] to and what you'll probably need to make sure your home is proper for you. I mean they didn't come until yesterday, so you know that's like 3–4 days after he gets home. What happens in those four days if the home is not proper for him?’

Caregivers were only sometimes approached to determine if they felt ready to handle their family member once discharged home. Several caregivers indicated that any sort of pamphlet or information guide would have been beneficial. The following interview shows the information need was so great that the caregiver used a roommate's information booklet:

Caregiver: ‘No booklet or pamphlet, but the people that were in the next bed got a booklet because they were scheduled for surgery … So they had the pre-op things. So I borrowed their booklet, took it home and copied it … I didn't know about how he was supposed to bend and not bend.’

Interviewer: ‘OK, and was the booklet helpful for you?’

Caregiver: ‘Yes, we still have it here.’

The associated field note highlights the resourcefulness of the caregivers, albeit at the risk of using inappropriate health care information.

Memo: August 19^th^, 2010

‘While conducting the interview this afternoon I got the impression that she was surprised that no information brochure was given. She did some problem solving and borrowed someone else's – she seemed to find this very helpful. They did understand that this booklet was for a [total] hip replacement rather than a hip fracture, but the patient and spouse felt it would be helpful to have a hip fracture booklet with similar information.’

Fortunately, the information in the booklet was applicable to the patient because the surgical repair was a hemi-arthroplasty, therefore needing the same safety measures and exercises that were outlined in the information booklet for elective total hip joint replacements.

The excerpt that follows is a representative of the concern that many caregivers expressed because they did not receive information about how the patient was doing and the associated stress:

Caregiver: ‘Apparently she went for some x-rays and some heart tests last week and we have never even gotten any results or nothing … and I'm about to ask about her medications that they are giving her - we don't know anything.’

Many patients and caregivers expressed the need for more information from the health care provider, in order to better assist the patient during recovery.

### Facilitators and barriers of information sharing

#### Facilitator: prior health care experience

Family caregivers who had prior health care knowledge were able to navigate the system much more easily than people who did not have this knowledge. Three of the family caregivers had previous health care experience and were at a noticeable advantage when taking care of their families. They were all retired nurses and were able to use their prior health care experience to help with rehabilitation, medication and system navigation.

Of the three caregivers who had prior health care system knowledge, two of them still struggled during the transition process. For example, one caregiver was very stressed when her husband returned home because she was unaware of the physiotherapy limitations he had, having not been not involved in his physiotherapy sessions at the hospital:

Interviewer: ‘Yeah, so you called for information.’

Caregiver: ‘Yes, I did. And I was letting them know I was a little concerned that he didn't seem to be getting as much physio as I thought would be in his best interest … But I don't know whether any precaution was really advised as far as the amount of flexion or anything … because it sounded like he would be able to lift and bend his knees you know as an exercise, but not by very much because the muscle and the incision needed to heal first too.’

The other retired nurse caregiver felt that her experience gave the health care providers confidence in her ability to care for her mother while at home:

Caregiver: ‘But Mom, I think they were satisfied in the fact that they knew that you had professional care at home … Other people aren't able to have that kind of care.’

When moving from the role of health care provider to the role of family caregiver, these caregivers still experienced the burdens that go along with caregiving regardless of the nursing experience they had.

#### Barrier: lack of a trusting relationship

The importance of trusting relationships between health care professionals and family caregivers at a critical time was especially noticeable during observations of situations where families lack knowledge of the health care system and are depending on health care providers to help make the best care decisions.

Memo: October 12^th^, 2010

‘After meeting with this family a few times now, it is easy to see how involved they are in caring for their mother. The son was becoming quite distressed while explaining the difficulties he has had to overcome. He felt that he was given the wrong information numerous times. He shared many stories where he felt he was misguided. He was let go from his job just days before. He felt the pressure as he was trying to deal with finding a home for his mother while packing up his office.’

Many of the caregivers were not happy with the information they received from health care providers and in some cases were told information that they later realised was not accurate, creating a lack of trust in their health care providers.

Caregiver: ‘So that was nobody's fault, so you know we went from thinking, you know, she'll be in there for a week and walking home in a week - that was July 17th. I don't think that's [walking] been very normal since … And that's not anybody's fault. I certainly, you know, I don't blame anybody for that, but a lot of the events that happened in between were certainly very questionable … along the way’.

Caregivers’ lack of trusting relationships with health care providers led to a lack of trust in the health care system as a whole:

Caregiver: ‘I think that the sorrowing part that I see, is that it's just all about money, that's all it seems to be … we probably have one of the best health care systems in the world but it's cracking at the seams and you can see it. If this experience taught me anything it was that our health care system is in jeopardy now.’

The lack of trusting relationships with staff prevented the caregivers from accessing the appropriate information needed during the rehabilitation journey. Their experiences revealed frustration, anxiety and anger over the lack of opportunity for meaningful information sharing.

One health care provider discussed how caregivers should be involved to ease confusion and to build a trusting relationship:

Healthcare provider: “Transitions are hard, and it's you know I think as somebody that could have a family member going through all the steps it would be very frustrating, but I find that if you give the information from outset, that this is, is what might happen and we'll work with you and, and discuss things as we go, as long as people are given options, and given all the information and also time to process the information, so that when their discharge comes, they're ready.

The statement above represents a dialogue that should happen between health care providers and family caregivers, but as seen in other excerpts, this is not happening consistently.

#### Facilitator and barrier: rural setting

The rural setting was both a facilitator and a barrier to information sharing. In some cases the health care providers in the rural setting have the advantage of knowing the community very well and are thus better able to advise patients and families about available community services, such as what the retirement homes/long-term care facilities offer, costs and other information:

Health care provider: ‘…because we're a small hospital… we know the staff, you get to know the patients. And a lot of us live in the community as well.’

For a family caregiver, the rural setting, which may translate into long distances to travel, can create added stress when attempting to help their family member's recovery. Distant families also experienced stress related to not knowing how the patient is doing, feeling unable to assist as much as they would like and depending on the telephone to communicate with their family member and health care providers.

Caregiver: ‘Yeah, we kept calling the hospital, and we were told that you know, she had indeed broken her hip, I believe it was later that evening, and they were expected to be doing surgery on her, but it ended up being the following day before they actually did the surgery on her, so it seemed like an awfully long time.’

Interviewer: ‘And how soon after that were you able to come and visit?’

Caregiver: ‘We couldn't come down until Saturday’

Memo: April 18^th^, 2010

‘We were at the hospital today conducting some follow-up interviews with two of our participants. We were observing the nursing station before we left for the day. A caregiver who didn't live close called to ask some questions about her mother. The nurse was quite abrupt with her and told her that she could not give her any information. We talked with the caregiver later who said she had to call the hospital a number of times before someone finally told her that her mother had broken her hip but would be fine. This was the daughter who was from a distance, had been the caregiver for the last 5 years for her mother.’

Another barrier of living in a rural community is the isolated and potentially high-risk situation for some patients when they are discharged home. For example, one health care provider stated:

Health care provider: ‘we can get into some nightmares, you know just because, OK, so they can't go home, they live alone, they can't go home, you know they're in the middle of nowhere, they have no transportation, families don't live close, it's a two storey old farmhouse, you know just those sorts of things.’

## Discussion

This study found that family caregivers are valuable sources of information and are involved in multiple roles throughout the recovery process for hip fracture patients, as also described by Nahm and colleagues [24]. Family caregivers communicate previous medical history and functional status to the health care team and can help engage the patient or communicate information back to the patient. Families can help motivate the patient to complete daily exercises and follow therapists’ orders when needed. When the decision to transition to a new care facility is being made, families are seen as an integral part in this process. Some family members in this study wished they could have taken part in the therapy appointments so they could learn how to better assist the patient once at home. Practical involvement such as this not only helps the patient recover, but also eases the stress of family members because they will feel more prepared to manage the patient's needs at home.

Hickey [25] acknowledged that care provided by the family may be enhanced if information is given to the caregiver in an understandable way. Consistent with this, caregivers in our study expressed a need for helpful information in the form of something as simple as a pamphlet. They stated that just a small brochure outlining what to expect following surgery, or describing the types of assistive devices they may need to rent for use at home, would be extremely helpful to plan ahead for transitions and discharges. Written information is often a useful format as caregivers may have trouble remembering all of the information that is shared with them. Contact information for their physician and/or nurse practitioner is also useful information. As a result of this study, the hospital administration worked with clinicians involved with the care of hip fracture patients to develop a hospital-specific information booklet for patients with a hip fracture – this booklet is now in routine use.

Some patients and families experienced a lack of trust in the health care providers and the health system which ultimately affected information exchange between them and their health care providers. Limiting the information provided to caregivers can create frustration and difficulty throughout the rehabilitation process. Although Canadian privacy laws may limit information sharing between health care providers and family caregivers, as found in other work of our group [26], this did not seem to be a major source of the challenges identified by our participants. Communication and information exchange between health care providers and caregivers was not routine. Health care providers sometimes involve family caregivers as a source of knowledge about the patient; however, when this does not occur, care providers may not receive the information they need. Frustration was expressed by many patients and families about having family meetings being scheduled at times that were inconvenient for family caregivers, an example of hospital-driven rather than patient-centred care. A shift to a patient/family-centred care approach would involve engaging the patient and family in decision-making throughout the entire care trajectory. Engaging patient and families in decision-making should be a consistent practice, with the expectation that this be documented in the patients’ record (preferably in electronic health information systems), and which is supported by practice guidelines and recognised in quality and accreditation standards.

Family members who are thrust into a caregiving role as a result of a hip fracture or other sudden crisis event are likely to find themselves in roles filled with stress and uncertainty [12, 27]. Caregivers who had no prior experience either working in the system or being a patient in the system had difficulty knowing which questions to ask of health care providers and were unsure what to expect during the rehabilitation journey. In this study, even caregivers with prior knowledge of the health care system struggled with navigating through care transitions. In order to facilitate better communication between caregivers and health care providers, building trusting relationships is essential. When health care providers are able to take time for mutual sharing of information with patients and their families, a trusting relationship is more likely to develop, leading to a less stressful and potentially more effective care experience [27].

This study has highlighted the importance of meaningful communication with patients and families in order to achieve successful care transitions. Consistent with suggestions by Leutz [28] and by Edgren and Bernard [29], we would argue that efforts to achieve integrated care systems require active engagement of patients and caregivers as full members of the care team. As Edgren and Bernard [29] point out, for integrated care to be achieved, the contributions of all care providers need to be integrated – and the user (patient) needs to be considered as a provider. For frail older patients, such as those who have suffered a hip fracture, family caregivers are also going to play key roles in providing care and support. Where disconnects continue to exist between care settings, care providers and information systems, patients and caregivers are the only common participants in information exchange and in integrated care.

This study found both advantages and disadvantages associated with living in a rural area. For health care providers, working in a rural hospital and community allows them to become familiar with the community and its available resources and residential accommodations. This can be of benefit when determining if a patient's residence would be suitable for them post-discharge and in sharing information about the facility with the patient and family. As well, the staff at smaller rural hospitals became familiar with patients on repeated admissions to hospital. Disadvantages of living in a rural community are felt most by family caregivers who have to drive greater distances to visit their family member. Using available telecommunications technology (e.g., Skype, telehealth) may facilitate communication with distant family members. During the rehabilitation journey, making use of the advantages of a rural community while minimising the disadvantages of geography and distance would contribute to improved information sharing and smoother care transitions.

Most family caregivers wish to be well-informed about, and actively engaged in, the care and rehabilitation of their family member. This may be especially true in relation to transitions to other care settings or for a return home. Family members can be considered a key part of a patient's ‘circle of care’, and as such, should be actively involved in supporting the decision-making process for the patient, and in both providing and receiving information relevant to the patient's current and future care. Strengthening the process of information sharing among patients, family caregivers and health care providers during care transitions could improve health outcomes for both patients and their caregivers, as well achieve system benefits such as reduced hospital readmissions. When everyone involved has the information they need and are involved in all aspects of the transition process, care transitions will be smoother, the quality of care better and the patient's recovery faster.

In some respects, the challenges that are associated with effective communication and information sharing in patient care are analogous to those experienced in translating research knowledge into practice. As with knowledge translation, effective strategies to improve information sharing and care continuity for older patients with chronic illness may be those that involve active facilitation [30] of an on-going partnership [31] that respects the knowledge of all those involved – including family caregivers (as with a broad conceptualisation of ‘evidence’ [32]) and considers the context [33] in which the information sharing takes place – in this case a small hospital in a rural area.

This study has several limitations. Data were collected in one rural region of Ontario and thus may not be generalisable for care transitions in other health care contexts. On the other hand, many of the themes were consistent with findings from our studies in other sites [5, 10, 13, 14] as well as the work of others [34, 35], with the addition here of the rural setting. Another limitation is that we were unable to interview a health care provider and/or a family caregiver for each patient in each setting. Also, recruitment of additional family members separated from patients by distance could have provided further views on care transitions in rural contexts. Our study was limited to hip fracture patients; while we believe the experience of hip fracture patients is likely reflective of many older patients with complex health conditions, further research is warranted on care transitions of patients with other chronic illnesses. Patients in this study were all English-speaking; we anticipate that patients with language or other communication barriers could face additional challenges when communicating with health care providers. It must be noted that while patients in Canadian health care system receive many services free-of-charge (home health care beyond that provided by publicly funded home care programmes, and retirement home accommodation are exceptions), the system imposes financial pressures on health care organisations to discharge patients as quickly as possible from acute or community care, which limit the opportunity for communication or orderly transition planning.

Ensuring that correct and adequate information is exchanged between health care providers and family caregivers is important during any care transition. This helps to ensure that the transitions between and within health care settings and home occur smoothly. This study specifically examined the role caregivers, in a rural health care setting, play in information sharing during care transitions following hip surgery. As has been found for patients with other chronic illnesses [36], caregivers experienced many challenges to effective communication and continuity of care yet could play important roles in improving patient care.

## Figures and Tables

**Table 1.**
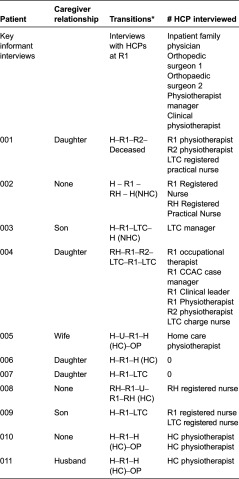
Participant characteristics

*H, Home; R1, rural hospital 1; R2, rural hospital 2; U, urban hospital; RH, retirement home; LTC, long-term care; NHC, no home care; HC, home care; OP, outpatient.

**Table 2.**
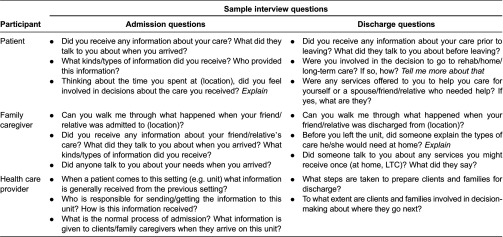
Sample interview questions
